# A phase 1b clinical trial of the multi-targeted tyrosine kinase inhibitor lenvatinib (E7080) in combination with everolimus for treatment of metastatic renal cell carcinoma (RCC)

**DOI:** 10.1007/s00280-013-2339-y

**Published:** 2013-11-05

**Authors:** Ana M. Molina, Thomas E. Hutson, James Larkin, Anne M. Gold, Karen Wood, Dave Carter, Robert Motzer, M. Dror Michaelson

**Affiliations:** 1 Memorial Sloan-Kettering Cancer Center, 1275 York Avenue, New York, NY 10065 USA; 2 Texas Oncology, Dallas, TX USA; 3 The Royal Marsden Hospital, London, UK; 4 Eisai Inc, Woodcliff Lake, NJ USA; 5 Eisai Ltd, Hatfield, Hertfordshire, UK; 6 Massachusetts General Hospital Cancer Center, Boston, MA USA

**Keywords:** Lenvatinib, Everolimus, Metastatic renal cell carcinoma, Antitumor, mTOR, VEGF

## Abstract

**Purpose:**

Lenvatinib is an oral multi-targeted tyrosine kinase inhibitor of VEGFR1-3, FGFR1-4, PDGFRβ, RET, and KIT. Everolimus is an oral mammalian target of rapamycin inhibitor approved for advanced renal cell carcinoma (RCC). This phase 1b study assessed safety, maximum tolerated dose (MTD), and preliminary antitumor activity of lenvatinib plus everolimus in metastatic RCC (mRCC) patients.

**Methods:**

Patients with advanced unresectable or mRCC and Eastern Cooperative Oncology Group performance status 0–1 were eligible (number of prior treatments not restricted). Starting dose was lenvatinib 12 mg once daily with everolimus 5 mg once daily administered continuously in 28-day cycles using a conventional 3 + 3 dose-escalation design. At the MTD, additional patients were enrolled in an expansion cohort.

**Results:**

Twenty patients (mean 58.4 years) received lenvatinib [12 mg (*n* = 7); 18 mg (*n* = 11); 24 mg (*n* = 2)] plus everolimus 5 mg. MTD was established as once daily lenvatinib 18 mg plus everolimus 5 mg. The most common treatment-related treatment-emergent adverse events (all dosing cohorts) were fatigue 60 % (Grade ≥3: 10 %), mucosal inflammation 50 %, proteinuria (Grade ≥3: 15 %), diarrhea (Grade ≥3: 10 %), vomiting (Grade ≥3: 5 %), hypertension, and nausea, each 40 %. In MTD and lowest-dose cohorts (*n* = 18), best responses of partial response and stable disease were achieved in 6 (33 %) and 9 (50 %) patients, respectively.

**Conclusions:**

Lenvatinib 18 mg combined with everolimus 5 mg was associated with manageable toxicity consistent with individual agents and no new safety signals. Observed activity warrants further evaluation of the combination in advanced RCC patients.

## Introduction

Vascular endothelial growth factor (VEGF)-mediated angiogenesis and mammalian target of rapamycin (mTOR)-mediated regulation of cell growth, cell proliferation, cellular metabolism, and angiogenesis have been identified as key factors in the development of renal cell carcinoma (RCC) [1, 2]. Several agents that inhibit the VEGF pathway have shown clinical benefit in metastatic RCC (mRCC), including sorafenib, sunitinib, axitinib, pazopanib, and bevacizumab (in combination with interferon-α) [3]. Everolimus and temsirolimus, both of which target the mTOR pathway, have also shown clinical benefit in mRCC [3]. In previously treated mRCC patients, everolimus demonstrated partial response (PR) rates of 1.8 %, with no complete responses (CRs), and overall survival of 14.8 months [4]. Although treatment with everolimus is considered a reference standard for previously treated RCC patients, as currently recommended by NCCN Clinical Practice Guidelines in Oncology (NCCN Guidelines^®^), tumor responses are generally low and transient in the majority of patients [5–7].

Tumors are believed to become resistant to therapy through feedback mechanisms that compensate for targeted inhibition [1, 8]. Upregulation of hypoxia-inducible factor 1 target genes, including VEGF, has been implicated in RCC [9, 10]. Additionally, genetic alterations leading to constitutive activation of the mTOR signaling pathway have also been implicated in RCC [11, 12]. Theoretically, a combination of agents targeting both VEGF- and mTOR-mediated pathways could simultaneously block two critical signaling pathways activated in RCC and potentially overcome an aspect of resistance to single-agent therapy [13]. Lenvatinib is an oral, multi-targeted tyrosine kinase inhibitor (TKI) of VEGF receptors (VEGFRs) 1-3, fibroblast growth factor receptors 1–4, platelet-derived growth factor receptor β, RET, and KIT [14]. In phase 1 and 2 studies, lenvatinib has demonstrated an acceptable toxicity profile and antitumor activity in patients with multiple solid tumors, including advanced RCC [15–19]. Data from in vitro binding assays show that lenvatinib binding specificity is mostly restricted to the receptor kinase domain of the kinome dendrogram [20]. The binding specificity of lenvatinib may be associated with less off-target toxicity, although this needs validation in clinical trials.

In a randomized, open-label, phase 1b/2 study, we evaluated the use of everolimus in combination with lenvatinib in RCC patients with unresectable or metastatic disease (ClinicalTrials.gov: NCT01136733). The primary objectives of the phase 1b component reported here were to determine the dose-limiting toxicities (DLTs), maximum tolerated dose (MTD), and recommended phase 2 dose for lenvatinib plus everolimus.

## Methods

### Patient eligibility

Patients were aged ≥18 years, with histologically confirmed and documented evidence of unresectable advanced or mRCC and disease progression after prior therapy targeting the VEGF domain. Two patients who had received no prior regimens were enrolled prior to a protocol amendment. Eligible patients had an Eastern Cooperative Oncology Group performance status of 0 or 1, adequately controlled blood pressure, adequate hematologic, hepatic, renal, and blood coagulation function, and internationalized normalized ratio (INR) of ≤1.5. There was no upper limit on prior therapies.

Key exclusion criteria included prior exposure to lenvatinib, discontinuation of prior TKI due to toxicity, known intolerance to rapamycins, therapy with an anticancer agent or major surgery within 21 days, or treatment with any investigational agent within 30 days. Patients with significant cardiovascular impairment, bleeding or thrombotic disorders requiring anticoagulant therapy and therapeutic INR monitoring, prolongation of QTc interval (>480 ms), untreated or unstable metastases to the central nervous system, urine protein ≥1 g/24 h, uncontrolled diabetes [fasting glucose >1.5 × upper limit of normal (ULN)], fasting total cholesterol >7.75 mmol/L, and fasting triglyceride levels >2.5 × ULN were also excluded.

The study was approved by Institutional Review Boards at each participating site and carried out in accordance with local Independent Ethics Committee standards, World Medical Association Declaration of Helsinki, and the International Conference on Harmonisation of Technical Requirements for Registration of Pharmaceuticals for Human Use guidelines. All patients provided written informed consent prior to participation.

### Study design

This was the phase 1b component of a multicenter, open-label, phase 1b/2 study. The phase 2 component of the study is ongoing. The study used a standard “3 + 3” dose-escalation scheme. Patients were treated in sequential cohorts of escalating doses of lenvatinib in combination with everolimus, each administered once daily in 28-day treatment cycles until disease progression, development of unacceptable toxicity, or withdrawal of consent. The initial dose of lenvatinib was 12 mg once daily in combination with everolimus 5 mg once daily (Cohort 1). Subsequent doses were lenvatinib 18 mg once daily with everolimus 5 mg once daily (Cohort 2) and lenvatinib 24 mg once daily with everolimus 5 mg once daily (Cohort 3).

DLTs were assessed during the first treatment cycle and were defined either as treatment-related failure to administer ≥75 % of the planned dosage of lenvatinib/everolimus combination or as specific common toxicity criteria (CTC) Grade ≥3 hematologic or nonhematologic toxicities considered to be at least possibly related to lenvatinib and/or everolimus therapy. A treatment cycle was defined as 28 days, and dosing was continuous. Hematologic toxicities were defined as Grade 4 neutropenia lasting at least 7 days, febrile neutropenia with neutrophils <1 × 10^3^/μL and a recorded temperature >38.5 °C, or Grade ≥3 thrombocytopenia with bleeding or lasting more than 7 days. Nonhematologic toxicities were defined as Grade 3 toxicities for >7 days (except Grade 3/4 hyperamylasemia or hyperlipasemia without pancreatitis); in addition, nausea, vomiting, or diarrhea had to persist at Grade 3 or 4 despite maximal medical therapy. If one of the first three patients enrolled within a cohort demonstrated a DLT, an additional three patients were enrolled into that cohort. If two or more patients demonstrated DLTs during the first 4 weeks of therapy in any cohort, dose escalation was halted and, if necessary, additional patients were enrolled to the next lower dose to achieve a total of six patients in that cohort.

MTD was defined as the highest dose level resulting in ≤1 of 6 DLTs. The definition of confirmed MTD was the highest dose level resulting in ≤1/3 of at least 10 patients experiencing DLTs during Cycle 1 or intolerable toxicities that could not be managed with dose interruption and/or reduction during Cycle 2. Once the MTD was established, the patient cohort at MTD was expanded and the MTD validated by assessing DLTs during the first cycle and intolerable toxicities (i.e., not manageable with dose interruption and/or reduction) during the second cycle of therapy.

Tumor measurements were assessed by clinical examination, computed tomography or magnetic resonance imaging, and bone scans and were based on investigator review data in conjunction with a radiologist using modified Response Evaluation Criteria in Solid Tumors, version 1.1 [21].

Tumor response assessments were conducted at baseline and then approximately every 8 weeks, and responses confirmed at a follow-up examination after ≥30 days. Tumor response was defined as CR, PR, stable disease (SD) (minimum duration from randomization to SD ≥7 weeks), or progressive disease (PD). Disease control rate (DCR) was defined as the percentage of patients with a best overall response of CR, PR, or SD. Durable SD was defined as SD ≥23 weeks.

### Statistical analysis

Cohorts of three to six patients each in phase 1b were considered adequate to evaluate initial safety assessments supporting dose escalation. A minimum of 10 patients was considered adequate to confirm the MTD of phase 1b. The safety analysis set included all patients who received at least one dose of the study drug and have at least one postbaseline safety evaluation and was the analysis set for all safety and efficacy evaluations.

Baseline and demographic variables, including age, gender, and race, were summarized using descriptive statistics. Objective response rate (ORR), DCR, and durable SD rate were calculated with exact 95 % confidence intervals (CIs) using the Clopper and Pearson method. All statistical analyses were performed using SAS^®^ software (SAS Institute, Cary, NC, USA).

## Results

### Patient characteristics

There were 20 patients recruited, dosed, and included in this analysis. Demographics and baseline characteristics for the study population are summarized in Table 1. Mean age of patients was 58.4 years (standard deviation, 6.29), with the majority (90 %) being younger than 65 years. All patients were Caucasian, 70 % were male, and 85 % had received at least one prior anti-VEGF therapy. The median number of prior therapeutic anticancer RCC regimens received was 1.5 (range 0–4).

**Table 1:** Patient demographics and baseline characteristics

	Total (*N* = 20)	Cohort 1	Cohort 2	Cohort 3
		Lenvatinib 12 mg + everolimus 5 mg (*n* = 7)	Lenvatinib 18 mg + everolimus 5 mg (*n* = 11)	Lenvatinib 24 mg + everolimus 5 mg (*n* = 2)
Age, year				
Mean (SD)	58.4 (6.29)	58.0 (3.92)	58.1 (7.97)	61.0 (2.83)
Median	59.0	59.0	58.0	61.0
Min, max	46, 72	53, 62	46, 72	59, 63
Age group, *n* (%)				
<65 year	18 (90)	7 (100)	9 (82)	2 (100)
≥65 year	2 (10)	0	2 (18)	0
Gender, *n* (%)				
Female	6 (30)	3 (43)	2 (18)	1 (50)
Male	14 (70)	4 (57)	9 (82)	1 (50)
White, non-Hispanic, *n* (%)	20 (100)	7 (100)	11 (100)	2 (100)
Number of prior anticancer regimens, median (range)^a^	1.5 (0–4)	3 (1–4)	1 (0–4)	0.5 (0–1)
Patients with prior anti-VEGF treatment, *n* (%)	17 (85)	7 (100)	9 (82)	1 (50)
Patients with prior mTOR-targeted therapy, *n* (%)	7 (35)	4 (57)	3 (27)	0
Patients with both prior anti-VEGF and mTOR-targeted therapy, *n* (%)	7 (35)	4 (57)	3 (27)	0

*mTOR* mammalian target of rapamycin, *SD* standard deviation, *VEGF* vascular endothelial growth factor

^a^Therapeutic regimens for RCC

### Duration of treatment, dose-limiting toxicities, and maximum tolerated dose

Median overall duration of treatment (range) was 19.0 (1–69) weeks across all dosing cohorts and was 32.0 (1–68), 16.0 (1–69), and 3.5 (2–5) weeks for Cohorts 1, 2, and 3, respectively. Median number of treatment cycles was 5.5 (range 1–18). Treatment was discontinued for reasons other than disease progression by 30 % (6/20) of patients: one (14 %) patient in Cohort 1 for reason listed as “other—clinical deterioration”; three (27 %) patients in Cohort 2 [two patients due to adverse events (AEs); one withdrew consent]; two (100 %) patients in Cohort 3 (one due to AEs; one due to patient choice). Lenvatinib doses were reduced in 10 patients (50 %) and treatment interrupted in 14 patients (70 %). No patients required a dose reduction in everolimus; however, dose interruptions of everolimus were needed for nine patients. The lowest-dose cohort had the fewest number of lenvatinib dose reductions (Cohort 1: 29 %; Cohort 2: 64 %; Cohort 3: 50 %); however, the number of patients with treatment interruption was comparable in the three cohorts (Cohort 1: 71 %; Cohort 2: 73 %; Cohort 3: 50 %).

Four patients experienced DLTs: (1) CTC Grade 3 abdominal pain (Cohort 1; DLT equivalent); (2) failure to administer >75 % of planned dose due to Grade 3 elevated creatinine phosphokinase, Grade 2 fatigue, and Grade 1 reflux (Cohort 2); (3) Grade 3 nausea and vomiting (Cohort 3; DLT equivalent); and (4) failure to administer >75 % of planned dose due to Grade 2 mucosal inflammation (Cohort 3). Cohort 2, lenvatinib 18 mg once daily and everolimus 5 mg once daily, was identified as the MTD.

### Safety

Twenty patients who received at least one dose of study therapy with ≥1 postbaseline safety evaluation were included in the safety population. Treatment-emergent AEs (TEAEs) occurred in 90 % (*n* = 18) of study patients, although the majority of these AEs were Grade 1/2. Grade 3/4 AEs were reported in 15 (75 %) patients. One patient experienced Grade 5 cholangitis (Cohort 1) deemed unrelated to study medication and occurred 11 days after the last lenvatinib dose.

The most common treatment-related AEs were fatigue (60 %), mucosal inflammation (50 %), diarrhea, hypertension, nausea, proteinuria, and vomiting (40 % each) (Table 2). The most common CTC Grade ≥3 treatment-related AEs were hypertriglyceridemia (15 %), proteinuria (15 %), diarrhea, and fatigue (10 % each) (Table 3). Hypertriglyceridemia and proteinuria occurred in 3/11 patients in Cohort 2 but was not present in Cohort 1 or 3.

**Table 2:** Treatment-related AEs occurring in ≥20 % of patients (safety analysis set)

	Total (*N* = 20)	Cohort 1	Cohort 2	Cohort 3
		Lenvatinib 12 mg + everolimus 5 mg (*n* = 7)	Lenvatinib 18 mg + everolimus 5 mg (*n* = 11)	Lenvatinib 24 mg + everolimus 5 mg (*n* = 2)
Patients with grades 1–5 AEs, *n* (%)	18 (90)	7 (100)	9 (82)	2 (100)
Grades 1–5 (≥20 %) AEs by patient count, *n* (%)				
Fatigue	12 (60)	4 (57)	6 (55)	2 (100)
Mucosal inflammation	10 (50)	3 (43)	5 (46)	2 (100)
Diarrhea	8 (40)	3 (43)	4 (36)	1 (50)
Hypertension	8 (40)	5 (71)	3 (27)	0
Nausea	8 (40)	4 (57)	3 (27)	1 (50)
Proteinuria	8 (40)	3 (43)	5 (46)	0
Vomiting	8 (40)	4 (57)	3 (27)	1 (50)
Decreased appetite	7 (35)	2 (29)	4 (36)	1 (50)
Rash	7 (35)	2 (29)	4 (36)	1 (50)
Constipation	5 (25)	2 (29)	2 (18)	1 (50)
Epistaxis	5 (25)	2 (29)	3 (27)	0
Hypertriglyceridemia	5 (25)	0	5 (46)	0
Edema peripheral	5 (25)	1 (14)	4 (36)	0
Dry skin	4 (20)	2 (29)	2 (18)	0
Dyspnea	4 (20)	1 (14)	2 (18)	1 (50)
Weight decreased	4 (20)	1 (14)	3 (27)	0

**Table 3:** Grade ≥3 treatment-related AEs in safety analysis set

	Total (*N* = 20)	Cohort 1	Cohort 2	Cohort 3
		Lenvatinib 12 mg + everolimus 5 mg (*n* = 7)	Lenvatinib 18 mg + everolimus 5 mg (*n* = 11)	Lenvatinib 24 mg + everolimus 5 mg (*n* = 2)
Patients with Grade ≥3 AEs, *n* (%)	14 (70)	5 (71)	8 (73)	1 (50)
Patients with Grade 5 AEs, *n* (%)	0	0	0	0
Grade 3/4 AEs by patient count, *n* (%)				
Hypertriglyceridemia	3 (15)	0	3 (27)	0
Proteinuria	3 (15)	0	3 (27)	0
Diarrhea	2 (10)	1 (14)	1 (9)	0
Fatigue	2 (10)	1 (14)	1 (9)	0
Abdominal pain	1 (5)	1 (14)	0	0
Anemia	1 (5)	1 (14)	0	0
Blood creatine phosphokinase ↑	1 (5)	0	1 (9)	0
Cardiomyopathy	1 (5)	0	1 (9)	0
Cellulitis	1 (5)	0	1 (9)	0
Edema peripheral	1 (5)	1 (14)	0	0
Ejection fraction ↓	1 (5)	0	1 (9)	0
Gastric hemorrhage	1 (5)	0	1 (9)	0
Gastritis	1 (5)	0	1 (9)	0
Hypercholesterolemia	1 (5)	0	1 (9)	0
Hyponatremia	1 (5)	0	1 (9)	0
Hypophosphatemia	1 (5)	1 (14)	0	0
Lipase ↑	1 (5)	0	1 (9)	0
Lung infection	1 (5)	0	1 (9)	0
Nausea	1 (5)	0	0	1 (50)
Vomiting	1 (5)	0	0	1 (50)
White blood cell count ↓	1 (5)	0	1 (9)	0

### Tumor response

**Fig. 1:**
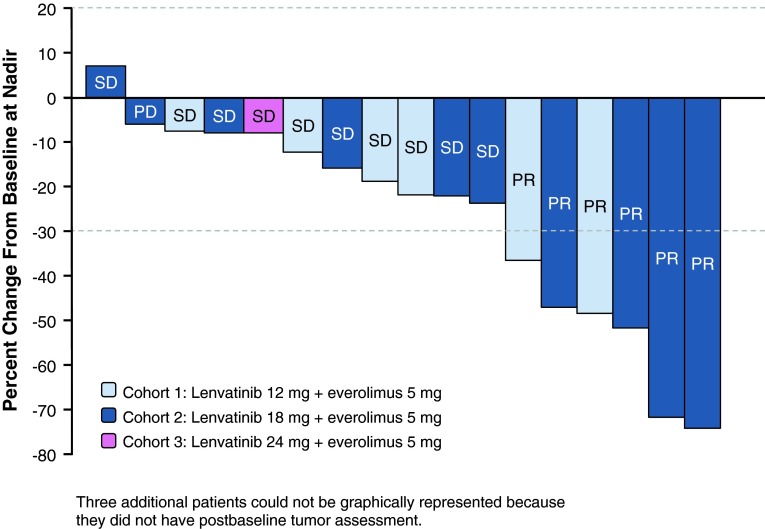
Waterfall plot of percent of maximum change in summed longest diameter of target lesion from baseline, by investigator. *PD* progressive disease, *PR* partial response, *SD* stable disease

**Fig. 2:**
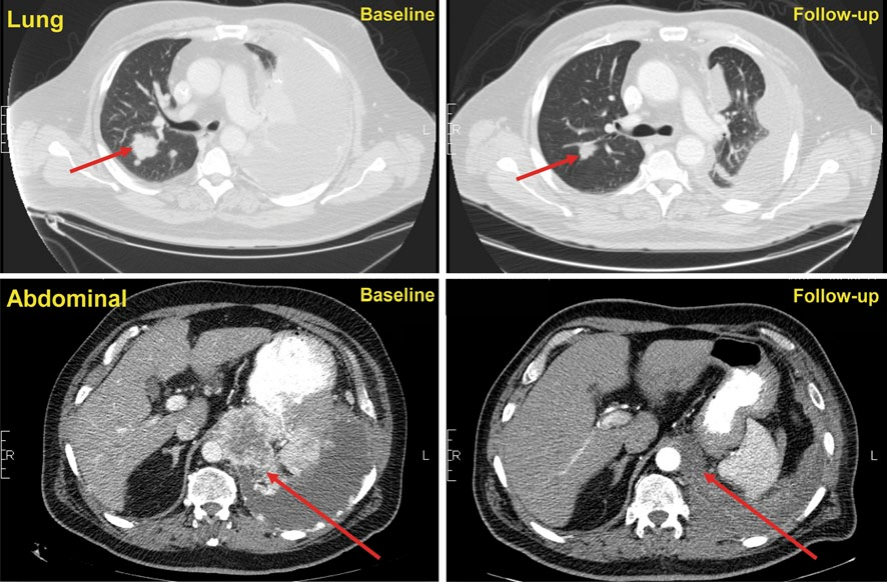
Radiologic response to lenvatinib in combination with everolimus. Baseline and follow-up images of one patient treated with lenvatinib 14 mg and everolimus 5 mg

At the data cutoff date, PR rate was 30 % (95 % CI 11.9–54.3, *n* = 6; Cohort 1, *n* = 2; Cohort 2, *n* = 4) as assessed by investigators. No CRs were observed (Table 4). In the MTD and lower-dose cohort, PR was observed in 6/18 patients (33 %) and SD or PR was achieved in 15/18 patients for a DCR of 83.3 % (Table 4). Four patients experienced durable SD. The median progression-free survival (PFS) was 330 days (95 % CI 157–446; approximately 10.9 months) at the MTD and lower-dose cohort, while the 6- and 12-month PFS rates were 72.1 % (95 % CI 48.8–95.4 %) and 49.5 % (95 % CI 22.7–76.2 %), respectively. A waterfall plot of maximum percent tumor change from baseline to postbaseline nadir is shown in Fig. 1. Figure 2 illustrates a patient’s radiologic response to combined therapy.

**Table 4:** Best response

Objective response	Total (*N* = 20)	Cohort 1	Cohort 2	Cohort 3
		Lenvatinib 12 mg + everolimus 5 mg (*n* = 7)	Lenvatinib 18 mg + everolimus 5 mg (*n* = 11)	Lenvatinib 24 mg + everolimus 5 mg (*n* = 2)
PR, *n* (%)	6 (30.0)	2 (28.6)	4 (36.4)	0 (0.0)
95 % CI	(11.9–54.3)	(3.7–71.0)	(10.9–69.2)	
SD (≥7 weeks), *n* (%)	10 (50.0)	4 (57.1)	5 (45.5)	1 (50.0)
95 % CI	(27.2–72.8)	(18.4–90.1)	(16.7–76.6)	(1.3–98.7)
Durable SD rate (SD ≥ 23 weeks), *n* (%)	4 (20.0)	2 (28.6)	2 (18.2)	0 (0.0)
95 % CI	(5.7–43.7)	(3.7–71.0)	(2.3–51.8)	
PD, *n* (%)	1 (5.0)	0 (0.0)	1 (9.1)	0 (0.0)
DCR (CR+PR+SD), *n* (%)	16 (80.0)	6 (85.7)	9 (81.8)	1 (50.0)
95 % CI	(56.3–94.3)	(42.1–99.6)	(48.2–97.7)	(1.3–98.7)
Unknown, *n* (%)	3 (15.0)	1 (14.3)	1 (9.1)	1 (50.0)
95 % CI	(3.2–37.9)	(0.4–57.9)	(0.2–41.3)	(1.3–98.7)

*CI* confidence interval, *CR* complete response, *DCR* disease control rate, *PD* progressive disease, *PR* partial response, *SD* stable disease

## Discussion

Therapeutic targeting of VEGF- and mTOR-mediated pathways has expanded available treatment options for mRCC [3]. Several challenges remain to improving targeted treatment outcomes once resistance to initial single-agent therapy arises. Optimal sequencing of these agents has not been defined, and clinical development of combinations of agents has been challenging due to unacceptable toxicity, complications of administration (e.g., combining oral and intravenous administration routes), or transient tumor responses. There is a continued need for more effective combinations of targeted agents, namely those associated with improved efficacy and manageable toxicity relative to single-agent sequential treatment [1, 3, 6].

The MTD and recommended phase 2 dose in this phase 1b component was confirmed to be lenvatinib 18 mg once daily in combination with everolimus 5 mg once daily. All patients were previously treated with therapeutic regimens for RCC (with the exception of two patients with no prior treatment who were enrolled prior to a protocol amendment). At the MTD and lower-dose cohort (lower-dose cohort: lenvatinib 12 mg plus everolimus 5 mg once daily), the combination of lenvatinib and everolimus was associated with manageable toxicity. Treatment-related AEs were consistent with class effects typical of VEGFR and mTOR inhibitors, with no new safety signals observed. The apparent lack of additive toxicity from the combination may have important clinical implications as unexpected, and severe toxicities observed with other combined treatments have thus far limited clinical development of effective regimens [22–24]. Fatigue, mucosal inflammation, proteinuria, hypertension, as well as gastrointestinal (GI) toxicity (nausea, diarrhea, and vomiting) were the most common AEs. Proteinuria, hypertriglyceridemia, diarrhea, and fatigue were the most common Grade ≥3 treatment-related TEAEs.

A phase 1 study of everolimus plus sorafenib was conducted in advanced mRCC patients [24]. While the PR rate of 25 % was greater than typically observed with either drug as monotherapy, dose reductions (*n* = 3 of eight patients in one cohort) or study discontinuation (*n* = 2) was necessary for GI toxicity, and there was a substantially higher incidence of rash (55 vs. 29 % for everolimus alone, 40 % for sorafenib alone) [24–26]. In a phase 2 trial combining full doses of bevacizumab with everolimus, median PFS and ORR in previously untreated and previously treated patients were 9.1 and 7.1 months (30 and 23 %), respectively [27]. The combination regimen was associated with a toxicity profile consistent with the known toxicities of each single agent, prompting an attempt to study this combination in a phase 3 setting; however, the phase 3 trial was closed prematurely due to failure of accrual. Additional phase 2 trials in mRCC using combinations of bevacizumab and temsirolimus [28] or bevacizumab and everolimus [29] were associated with prohibitive toxicity and low clinical activity to warrant consideration as a treatment regimen, which has raised serious questions as to whether targeted agents in RCC can be dosed fully together as combination treatment.

Whether the responses observed with the present combination therapy confer an advantage relative to single-agent therapy will require further study. In our exploratory analysis of tumor response, treatment at or below the MTD resulted in a PR rate of 33.3 %, durable SD (≥23 weeks) rate of 22.2 %, and median PFS of approximately 10.9 months in patients who received a median of 1.5 prior anti-VEGF treatments. Anti-VEGFR multi-TKI treatments currently available for previously treated mRCC patients include sorafenib, sunitinib, axitinib, and pazopanib and have demonstrated response rates ranging between 9 and 34 % and PFS between 4.7 and 8.3 months as single-agent treatment [30–34]. Everolimus 10 mg once daily treatment of patients with mRCC who had progressed on sunitinib, sorafenib, or both resulted in a PR rate of 1.8 % and a median PFS of 4.9 months [4]. Although data from different clinical trials must be interpreted with caution, the response rate of 33 % and PFS of 10.9 months for lenvatinib in combination with everolimus are encouraging in the context of what has been reported in the literature.

In conclusion, the MTD and recommended phase 2 dose was identified to be lenvatinib 18 mg once daily plus everolimus 5 mg once daily. Most treatment-related AEs were consistent with class effects typical of mTOR and VEGFR inhibitors and were managed effectively by dose interruptions or reductions. Importantly, no new safety signals were evident with the use of combination therapy. The clinical benefit rate, including PRs and durable SD, appears favorable, and the ongoing phase 2 portion of this study will further elucidate the clinical value of this regimen.
